# Sustained-Release Fillers for Dentin Disinfection: An Ex Vivo Study

**DOI:** 10.1155/2019/2348146

**Published:** 2019-05-22

**Authors:** Bernhard Funk, Sharonit Sahar-Helft, David Kirmayer, Michael Friedman, Doron Steinberg

**Affiliations:** ^1^The Institute of Dental Sciences, Faculty of Dental Medicine, The Hebrew University of Jerusalem, Jerusalem, Israel; ^2^Department of Endodontics, Faculty of Dental Medicine, The Hebrew University of Jerusalem, Jerusalem, Israel; ^3^The Institute of Drug Research, School of Pharmacy, Faculty of Medicine, The Hebrew University of Jerusalem, Jerusalem, Israel

## Abstract

*Enterococcus faecalis* is the most commonly recovered species from failed root canal treatments. In this study, we tested the capability of a novel intracanal sustained-release filler (SRF) containing cetylpyridinium chloride (CPC) to disinfect dentinal tubules of segmented human tooth specimens. Human dental root specimens were infected with *E. faecalis* V583 for 3 weeks in a static environment. The tested intracanal medicaments were SRF-CPC and calcium hydroxide (CH). Each medicament was introduced into the canal of the dental specimen and incubated for 7 days. The bacteriological samples were taken by shaving the dentine surrounding the root canal with dental burs ranging in size from ISO 014-020. The obtained dentine powder was collected in test tubes containing phosphate-buffered saline, sonicated, and plated on agar plates. Colony-forming units were counted after 48 h of incubation. Random specimens were also examined under confocal laser scanning microscopy and scanning electron microscopy. A statistical difference was found in the bacterial counts obtained from all layers of infected dentin between the control and the SRF-CPC groups. CH reduced bacterial viability significantly only in the first layer of the infected dentin, up to 150 *μ*m into the dentinal tubules. CLSM images showed that SRF-CPC killed most bacteria throughout the infected dentin up to 700 *μ*m of penetration. SEM images demonstrated the adhesion ability of SRF-CPC to the dentinal wall. In conclusion, SRF-CPC is a potential intracanal medicament for disinfecting dentinal tubules.

## 1. Introduction

According to Versiani and Ordinola-Zapata [1], the primary goal of a root canal treatment (RCT) is the removal of all microorganisms from the inner surface of the root canal system, to prevent reinfection and to establish or maintain healthy periapical tissues. Modern techniques and equipment have greatly contributed to the increase in clinical success rates and significantly shorten the time needed to complete the RCT, but there are still limitations to endodontic disinfection; viable biofilm cells can persist in undertreated and untreated locations of the root canal system due to the inherent challenges associated with their complex anatomy [2–4]. Dentinal tubules beneath endodontic biofilms are often invaded by bacterial cells [5].

The use of intracanal medication is a widely spread clinical practice to attain disinfection and prevent reinfection between appointments [6]. Ideally, an intracanal medicament should eliminate any remaining bacteria, reduce inflammation of periapical tissues, render canal contents inert and neutralize debris, act as a barrier against leakage from temporary filling, and help to dry persistently wet canals [7, 8]. Calcium hydroxide (CH) has good physical, biological, and pharmaceutical properties and is the most commonly used intracanal medicament. However, it is not very effective against resilient intracanal microorganisms as *Enterococcus faecalis*, the most commonly recovered species from failed RCTs [9]. In such cases, the obturating material has to be removed, followed by an irrigation protocol that comprises the use of sodium hypochlorite and chlorhexidine to effectively eradicate *E. faecalis*. Alternatively, or complementarily, an adequate intracanal medicament could be placed into the root canal to remove persistent *E. faecalis* cells and to disinfect the dentinal tubules.

The active pharmaceutical ingredient tested in this study was cetylpyridinium chloride (CPC). It is a broad-spectrum antiseptic commonly found in mouthwashes and is also used topically for minor infections of the mouth and throat [10, 11]. CPC is effective against Gram-positive bacteria, viruses, and fungi, presenting some gaps against Gram-negative bacteria [12–14]. For endodontic applications, CPC was already tested as a component of dental sealers, gutta-percha points, or irrigation solution; in all cases, it proved to be very effective against *E. faecalis* [15–17]. *E. faecalis* is among the increasing number of bacteria resistant to antimicrobial agents, which has become a worldwide health threat in the last decade [18, 19]. A recent study showed that repeated exposure of *E. faecalis* to chlorhexidine led to its resistance, whereas CPC did not elicit such response [20].

However, good antibacterial effects of a drug alone are not enough when combating endodontic microbiota that form biofilms. The penetration of the drug into the deep layers of the biofilm is limited by the biofilm matrix and diffusion is retarded [21, 22]. Nevertheless, higher concentrations of antimicrobial agents at the outer layer of the biofilm should increase the overall diffusion rates into the deeper layers [23].

To achieve high local concentrations over an extended period of time, a drug has to be incorporated into a delivery system that would prolong its release. The efficacy of CPC against dental plaque, as a component of sustained-release films, was already confirmed in a clinical study published by Friedman et al. [24]. In this study, we aimed to develop a sustained-release filler (SRF) for the root canal, supplemented with CPC, that presents good injectability and solidifies very quickly upon contact with an aqueous medium. In this study, SRF-CPC's disinfection power was tested on standardized human root canals, infected with vancomycin-resistant *E. faecalis*.

## 2. Materials and Methods

### 2.1. Preparation of the SRF

The detailed preparation procedure is set forth in the “Supporting Information” file. Briefly, the heat-sterilized polymer (Eudragit® RL) was dissolved in sterile-filtered *N*-methyl pyrrolidone-water mixture with CPC and a small amount of calcium chloride. The resulting formulation contained 0.5% of CPC.

### 2.2. Tooth Preservation and Ethical Approval

A total number of 17 extracted permanent single-rooted human teeth were preserved in a 0.01% w/v thymol solution at 4°C until use. Teeth were extracted for periodontal reasons. Ethical approval for experimental use was obtained in accordance with the Helsinki principles (Approval number: 0406-17-HMO).

### 2.3. Preparation of Dental Root Segments

The method was performed similar to the protocol used by Heling et al. [25], with some modifications. In short, dental roots were decoronated 1 mm below the cementoenamel junction using a diamond disc at low speed under water irrigation. Next, root segments were obtained by cutting 4 mm below the coronal end of each decoronated specimen. The main root canal of the segmented roots was standardized to an ISO size #011 using a gates glidden bur (GG) #4 (MANI, INC., Utsunomiya, Tochigi, Japan) at low speed. Standardized root segments were sonicated in water, sodium hypochlorite (5.25%), 17% ethylenediaminetetraacetic acid (EDTA), and again in water, each for 5 min. Root segments were then autoclaved for 15 min at 124°C and placed together inside a 50 ml test tube containing 10 mL of brain heart infusion broth (BHI; Neogen Corporation, Lansing, Michigan, USA) overnight to confirm sterility.

### 2.4. Infection of Standardized Root Segments

*Enterococcus faecalis* V583 stock cultures were used to prepare the working culture. *E. faecalis* was diluted in BHI and incubated overnight at 37°C under aerobic conditions in an orbital shaker. The overnight culture was diluted in BHI and adjusted to the 0.5 McFarland standard. Aliquots of 3 mL of the adjusted bacterial culture were added to the wells of a 12-well tissue culture plate (TCP), each one containing five segmented root specimens. The TCP was incubated at 37°C in aerobic conditions for 3 weeks. Bacterial broth medium was renewed every other day, and the purity of the culture was assessed in every medium change by optical microscopy by analyzing the morphological properties of the bacteria. Bacterial viability was evidenced by the visible increase in turbidity of the sterile broth medium after 24 hours following each renewal.

### 2.5. Disinfection of Root Canals by SRF-CPC and CH

Tooth segments were removed from the media and placed on the surface of 1 mL of 5% agar-agar inside a 12-well plate. Aliquots of 0.5 mL of 5% agar-agar were then added around each tooth specimen for stabilization during the following week. The presence of agar at the apical opening served to start the solidification of SRF-CPC. The canals were then filled with either SRF-CPC, calcium hydroxide (CH; Metapaste, Meta Biomed., LTD., Cheongju-si, Korea), or sterile water. Excess of the material was removed with a sterile spatula. The TCP was than inserted into a plastic flask. After one week of incubation with the corresponding filling material, root segments were submitted to colony-forming unit (CFU) analysis, confocal laser scanning microscopy (CLSM), and scanning electron microscopy (SEM).

### 2.6. CFU Analysis of Bacteria-Infected Dentin

**Figure 1:**
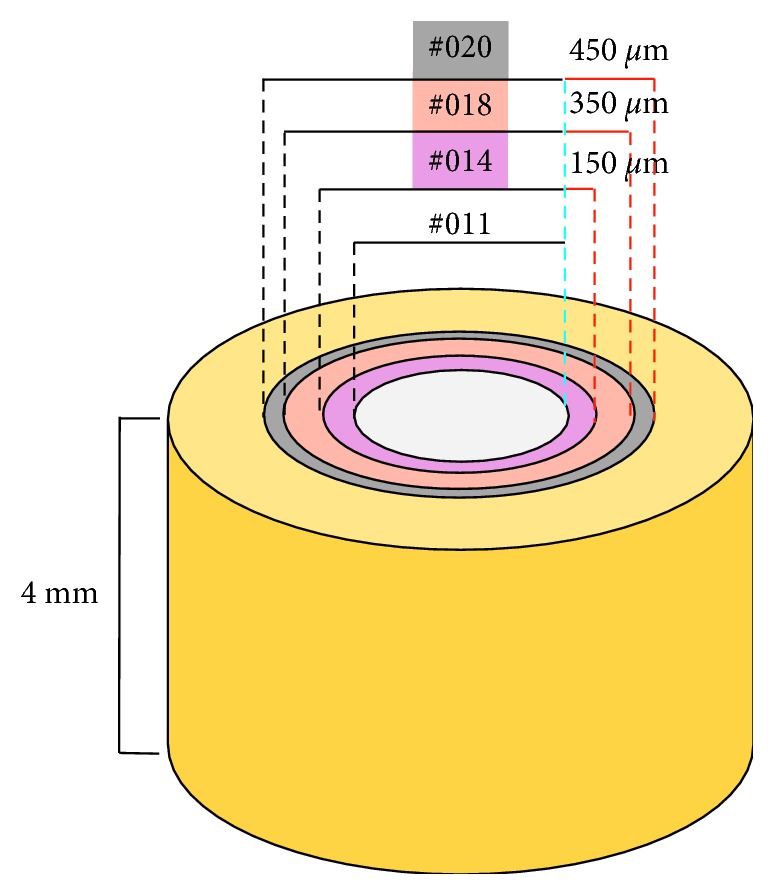
Dentin depth. The white circle represents the root canal prepared with an ISO size #011 bur. Colored circles represent different dentin layers at increasing depths, sampled by incrementing bur sizes (ISO #014, #018, #020). Red solid lines represent dentin depths covered by each bur, starting from the root canal wall (dashed blue line). Dentin depth = (Ø bur − Ø root canal)/2.

Sampling was performed as follows. First, the intracanal medicaments and biofilm were completely removed with an ISO #013 round carbide bur (THOMAS, Bourges, Cedex, France). Then, burs of increasing sizes (ISO #014, #018, #020; THOMAS) were used to obtain dentinal chips from different dentin layers. The dentin depth reached during the circumferential shavings of each bur corresponds to 150 *μ*m, 350 *μ*m, and 450 *μ*m, for every bur, respectively. See a schematic representation in Figure 1. Dentinal chips from each layer were collected in 2 mL Eppendorf tubes containing 1 mL of BHI. The tubes were sonicated for 5 min at 3500 kHz (D-7700 SINGEN/Htw., Elma Schmidbauer GmbH, Singen, Germany). Aliquots of 50 *μ*L of each sonicated sample were serially diluted and plated in BHI agar plates, incubated at 37°C overnight in aerobic conditions, and then photographed and counted using Digimizer Software (v4.6.1, MedCalc Software).

### 2.7. CLSM

The following steps were performed similar to the protocol used by Giardino et al. [26]. In short, root segments were longitudinally cut in the middle with a diamond saw and immediately placed into phosphate-buffered saline (PBS). Next, the half segments were immersed in 17% EDTA for 5 min and then washed in sterile DDW. Half roots were then immersed in 300 *μ*L of a mix of SYTO 9 and PI during 30 min, followed by a rinse in PBS for 1 min to remove the excessive stain. Specimens were immediately examined through a 10x magnification objective (EC Plan-Neofluar 10x/0.30 M27, Carl Zeiss Microscopy, LLC, United States) with an LSM 710 Axio Observer Microscope (Carl Zeiss Microscopy, LLC, United States). Excitation and emission wavelengths were 480/500 nm for SYTO 9 and 490/635 nm for PI, respectively.

### 2.8. SEM

To visualize the relation between the intracanal medicaments and the dentin surface of the root canal wall, split root segments underwent SEM. A modified version of the protocol employed by Brandwein et al. [27] was used. Briefly, the split root segments were immediately stored in 4% glutaraldehyde. Before the SEM, the specimens were incubated in a laminar flow hood for 4 h until the specimens were completely dry. SRGs were then mounted on a metal stub and sputter-coated with gold prior to SEM analysis. A high-resolution scanning electron microscope (Magellan XHR 400L, FEI Company, Netherlands) was used to examine randomly selected positions of each specimen next to the root canal wall.

### 2.9. Statistical Analysis

Data from the CFU analysis were transformed using the equation “*Y* = Log 2(*Y* + 1)” and then statistically analyzed with two-way ANOVA, followed by a post hoc Tukey's test.

## 3. Results

### 3.1. CFU Analysis Proves Dentinal Disinfection Capabilities of SRF-CPC

**Figure 2:**
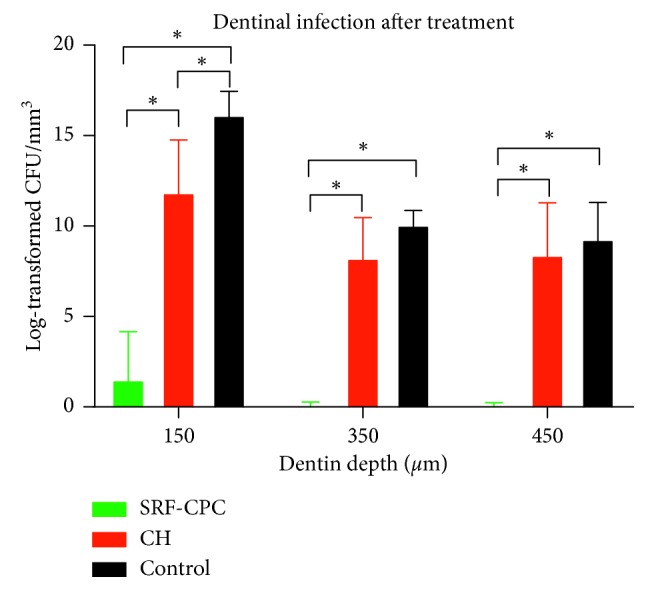
SRF-CPC's efficacy against *E. faecalis* inside dentinal tubes at different dentin depths. Equation *Y* = Log 2(*Y* + 1) was used for data transformation. Data are expressed as mean ± SD. ^*∗*^ *P* < 0.05, *n* = 3.

SRF-CPC disinfected dentinal tubules up to a distance of 450 *μ*m from the root canal wall. In one of the specimens exposed to SRF-CPC, few viable cells were found in the first sampled layer. Calcium hydroxide had some antibacterial effect, which was statistically significant only in the first layer, close to the root canal wall. Nevertheless, a slight tendency of reduction in comparison to the control can be observed for all penetration distances (Figure 2). SRG-CPC's antibacterial effects were statistically significantly higher than CH at all penetration levels.

### 3.2. CLSM Images Confirm Efficacy of SRF-CPC

**Figure 3:**
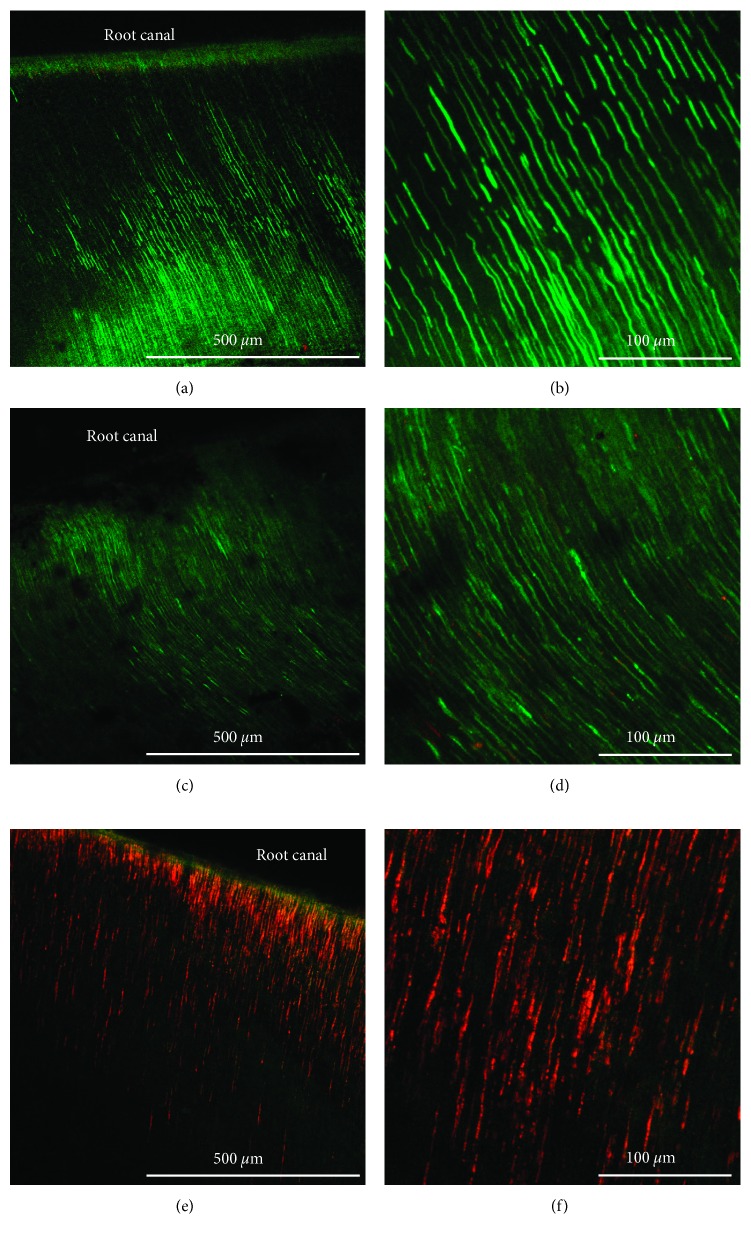
CLSM images of preinfected dental specimens after the corresponding treatment. (a, b) Negative control specimen; (c, d) specimen exposed to CH; (e, f) specimen exposed to SRF-CPC. Pictures in the right column were taken at higher magnification (30x). Red color corresponds to dead bacterial cells and green to viable cells.

Live bacterial cells, represented in green, were observed alongside the dentinal tubules throughout the whole length of the CH and control sample groups. Few dead cells (red color) were observed in the CH group, mainly close to the root canal wall. Exposure to SRF-CPC resulted in considerable amounts of dead bacteria inside the dentinal tubules, up to a distance of 700 *μ*m from the root canal wall. Some live bacteria were observed up to 300 *μ*m into the dentinal tubules from the root canal walls. Selected images are shown in Figure 3.

### 3.3. SRG-CPC Adheres to Dentin Surfaces

**Figure 4:**
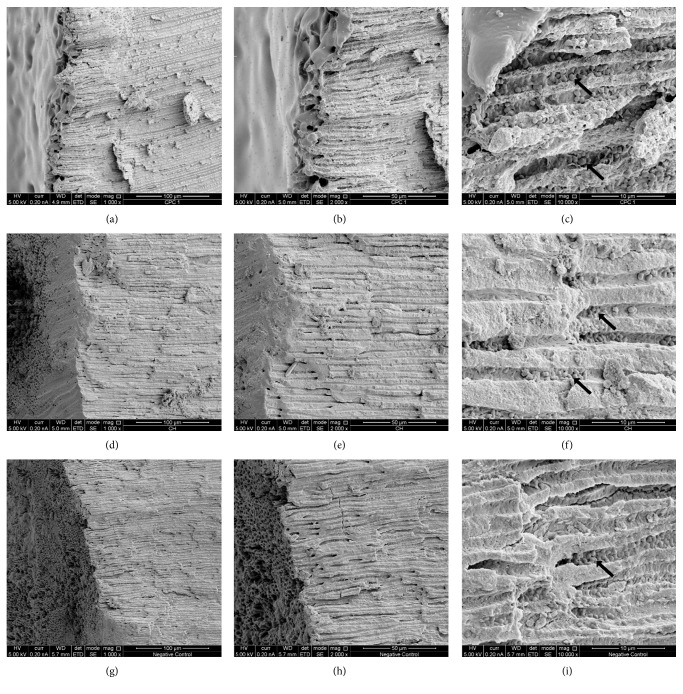
SEM images of dental specimens after intracanal treatment. SRF-CPC sample at 1000x (a), 2000x (b), and 10000x (c). CH sample at 1000x (d), 2000x (e), and 10000x (f). Negative control sample at 1000x (g), 2000x (h), and 10000x (i). Black arrows point at bacterial cells inside dentinal tubules.

Figure 4 shows SEM images of dental specimens exposed to intracanal medicaments in comparison to the control specimen. SRF-CPC adhered to the dentin and covered and sealed the openings of the dentinal tubules (Figures 4(a) and 4(b)). CH also covered the root canal wall, but not as homogeneous as SRF-CPC (Figures 4(d) and 4(e)). Dentinal tubules are completely open and exposed to the main root canal in the negative control sample (Figures 4(g) and 4(h)). SEM images of all specimens confirm successful bacterial infection of the dentinal tubules (Figures 4(c), 4(f) and 4(i)). The morphology of the observed bacteria confirms a monospecies infection with *E. faecalis*.

## 4. Discussion

In our study, dental root specimens were infected under static conditions for 3 weeks. Infection in a static environment for several weeks is a proven and simple method to achieve deep bacterial penetration into the dentin [28]. Other infection methods start with a centrifugation step [29, 30], which reduces the overall time required to infect the specimens, thus also reducing the risk of contamination during the infection period. Infection methods using the centrifugation step require the root specimens to be split in half before infection. For our experimental setup, the required shape of the dental root specimens needed to be cylindrical to allow the use of dental burs in the sampling process with precision. Hence, the static infection environment was chosen.

SRF-CPC was very effective in the disinfection of dentin up to at least 450 *μ*m from the root canal walls, as evidenced by the CFU counts. Concerns could be raised regarding the carryover effect of the tested antimicrobial contained in the sampled dentin debris [31, 32]. Even though we did not inactivate the CPC chemically, we removed the entire controlled-release delivery system off the main root canal with a bur sized larger than the root canal diameter. This allowed us to eliminate completely the intracanal residues of the tested medicaments before starting the sampling process, thus preventing carrying over remainders of the intracanal medicament into the sample suspensions that were plated on the agar plates. Nevertheless, diffused CPC molecules could have persisted inside the dentinal tubules. However, the minimum inhibitory concentration (MIC) of CPC for *E. faecalis* is very close to its minimum bactericidal concentration (MBC), 0.00031% vs. 0.000625% (data not shown), differing by a dilution factor of only 2. Considering that only a few micrograms of dentinal debris were diluted in 1 mL of BHI, equivalent to many 10-fold dilutions, even if there were carryovers, the concentrations inside the dentinal tubules must have been bactericidal in all cases where no growth was observed on the agar plates; therefore, the results do not present a false-negative outcome. Furthermore, the provided CLSM images of exposed dentinal tubules from specimens infected and medicated in the same way as the specimens used for the CFU experiment unequivocally demonstrate that the bacteria were dead in the dentinal tubules prior to CFU counting. CLSM analysis confirmed the efficacy of SRF-CPC and its ability to kill bacterial cells located even at 700 *μ*m of penetration distance, which corresponded to the depth of the infection. Few viable cells were observed, most of them located towards the root canal wall. The significant antibacterial efficacy of SRF-CPC is most likely related to the sustained release of the CPC from the SRF, in addition to the high sensitivity of *E. faecalis* to CPC [17]. Furthermore, the sealing of the dentinal tubules by the SRF might have caused additional environmental stress to bacteria residing deep inside the tubules [33]. An intimate relation between the SRF and the root canal walls was confirmed using SEM.

CH had very little effects on the viability of *E. faecalis* cells. This observation is in line with the findings of Heling et al. [34] who described similar results. They reported that CH did not inhibit bacteria from infiltrating the dentinal tubules and was unable to kill them after 24 h and 48 h and 7 days of incubation. Similarly, Siqueira and de Uzeda [35] reported that CH/saline solution paste was ineffective in disinfecting dentinal tubules infected with *E. faecalis* after 1 week of medicament exposure. Heling et al. [34] suggested that the dehydration of CH under their specific experimental conditions could have contributed to its inefficacy, which was not the case in our experiment since the dentinal blocks were maintained in a 100% relative humidity environment. In our study, dentinal specimens were placed on an agar-agar surface to achieve solidification of the injected SRF at the bottom end of the root segment, thus avoiding its leakage beyond the root canal. The diameter of the standardized root canals was 1.1 mm. This allowed for a small contact area between the intracanal medication and the agar, which could have caused some buffering of the high alkaline pH created by CH, thus reducing its antibacterial efficacy [36]. Nevertheless, CH was found to be ineffective against *E. faecalis* even in *ex vivo* experiments where its pH was not affected by the experimental conditions external to the dental specimen [35]. The lack of antibacterial efficacy of CH observed in our experiments can be explained by the ability of *E. faecalis* to resist alkaline stress [37] and the buffering property of hydroxyapatite constituting the dentinal tubules [38].

SRF-CPC quickly solidifies after injection when coming in contact with the humidity present on the internal walls of the root canal or at the apical end, thus preventing unwanted leakage. In a clinical setting, SRF-CPC is expected to stop penetration of periapical fluids into the root canal through the apical foramen, also acting as a physical and pharmaceutical barrier against leakage coming from a poor temporary obturation at the coronal end. Removal of SRF-CPC from the root canal after finishing the medication period is expected to pose little or no problem due to its soft consistency.

Intracanal medicaments are used in RCT with the aim of restricting bacterial regrowth and supplying continued disinfection inside the root canal [39]. Ideally, such medicaments should deliver and maintain significant concentrations of antimicrobial agent during the time period between RCT sessions; this is the role that the sustained-release formulations can readily play. Sustained-release delivery systems proposed for intracanal use range from preformed devices loaded with CHX [34, 40] to systems comprising micro- and nanoparticles [41, 42]. The formulation of SRF tested here contains Eudragit® RL polymers in approved solvents that provide good injectability into the root canal. Addressing concerns regarding the safety of the polymers composing the matrix of the SRF, it was proven that ammoniomethacrylate polymers favor cell growth on their surface which was addressed by Grin et al. [43]. We choose CPC as an active therapeutic agent, whereas many dental sustained-release delivery systems use CHX. As mentioned earlier, *E. faecalis* was found to develop resistance to CHX but not against CPC [20]. Furthermore, it was shown recently that CPC, in contrast to CHX, inhibits binding of lipopolysaccharides to toll-like receptors 4 involved in the inflammatory response [44]. As with any antiseptic, also the use of CPC may raise a question of possible cytotoxicity. The general outnumbering principle of antiseptic use, which stipulates that when killing one tissue cell, the tissue has one cell less, but when killing one bacterium cell, the bacterium is dead, generally proves true, which leads the regulators already over two decades ago to believe that antiseptics, inter alia CPC, are safe for use on oral mucosa [45]. Yet, the cytotoxicity of CPC cannot be completely ignored. Depending on the tested cell type, the half-population cytotoxic concentration of CPC (CC_50_) was found to vary between 0.003 and 0.001% [14, 46]. Tomino et al. [17] reported the MIC of CPC against *E. faecalis* to be 0.0001%. This means that there is a window of effective concentrations between 3 × 10^−4^% and 10^−3^% before any cytotoxic effect could even potentially be seen. Due to flexibility of the pharmaceutical formulation, the concentration of CPC released in practice could be adjusted to minimize side effects. Regarding the polymeric matrix of the SRF-CPC, it was demonstrated that ammoniomethacrylates polymers USP, whereto Eudragit® RL pertains, are capable of promoting cell growth on surface, in particular the adhesion, proliferation, and differentiation of human mesenchymal stem cells [43]. This leads us to believe that SRF-CPC is safe; however, more studies are required to confirm its innocuity before clinical use.

The superior dentinal disinfection capability of SRF-CPC over CH against vancomycin-resistant *E. faecalis* is clearly visible. Since CH is still the standard intracanal medicament, it was used for comparison in this study. CH might be the appropriate medicament in most clinical cases; however, when confronted with failed RCT due to the presence of *E. faecalis*, a different approach is necessary.

## 5. Conclusion

SRF-CPC is a potential intracanal medicament for root canal disinfection.

## Data Availability

The data (raw image files and CFU counts) used to support the findings of this study are available as supplementary materials.

## Supplementary Materials

Supplementary Materials Preparation of SRF-CPC.
