# Corrigendum to “Sustained-Release Fillers for Dentin Disinfection: An Ex Vivo Study”

**DOI:** 10.1155/2022/9754035

**Published:** 2022-10-25

**Authors:** Bernhard Funk, Sharonit Sahar-Helft, David Kirmayer, Michael Friedman, Doron Steinberg

**Affiliations:** ^1^The Institute of Dental Sciences, Faculty of Dental Medicine, The Hebrew University of Jerusalem, Jerusalem, Israel; ^2^Department of Endodontics, Faculty of Dental Medicine, The Hebrew University of Jerusalem, Jerusalem, Israel; ^3^The Institute of Drug Research, School of Pharmacy, Faculty of Medicine, The Hebrew University of Jerusalem, Jerusalem, Israel

In the article titled “Sustained-Release Fillers for Dentin Disinfection: An Ex Vivo Study” [1], the corresponding author's e-mail address was given incorrectly. The correct e-mail address is bernhard.funk@mail.huji.ac.il, as shown above.
