# Supplementary material for: Sustained-Release Fillers for Dentin Disinfection: An Ex Vivo Study
Source: Int J Dent. 2019 May 22;2019:2348146. doi: 10.1155/2019/2348146 (PMC6556298; doi:10.1155/2019/2348146)
Supplement: Supplementary Materials — Preparation of SRF-CPC. [file 2348146.f1.zip › 2348146.f1/Supporting information.docx]

**Preparation of SRF-CPC**

We obtained Eudragit® polymers as free samples from Israeli representatives of Evonik® (Wilhelm Rosenstein Ltd, Herzlia, Israel). Specifically, Eudragit® L and RL were used, corresponding to polymethacrylates type A, USP (i.e. as defined in the United States Pharmacopeia), and ammoniomethacrylates type A, USP. Calcium chloride was obtained from Merck, Israel; cetylpyridinium chloride (CPC), and N-methyl pyrrolidone (NMP) were obtained from Sigma-Aldrich, Israel; water was used purified from ion-exchange column and further redistilled.

The SRGs were prepared as follows: calcium chloride was dissolved in minimum amount of water and diluted with about 80% of the required volume of N-Methyl-2-pyrrolidone (NMP). Next polymers were added while vigorously mixing with a Vortex® mixer and placed for about 15 minutes into an oven heated to 40°C, until complete dissolution of the polymers. Thereafter, CPC was added to the solution using Vortex® mixer, and the remainder of the solvent was added to facilitate dissolution. The solution was left standing at 40°C for another 15 minutes to ensure complete dissolution and was stored at room temperature until used. For the aseptic preparation, the weighed amount of polymers was dissolved in ethanol and cast onto Petri dishes in an aseptic environment; the solution of CPC and calcium chloride in NMP with minimum amount of water was sterile-filtered using a 0.22 μm Nylon® filter into a sterile vial containing the dried polymer aseptically cut into small pieces. The mixture was vigorously mixed and placed in an oven at 40°C for about 30 minutes to complete dissolution.
